# Schistosomiasis Screening of Travelers to Corsica, France

**DOI:** 10.3201/eid2201.151606

**Published:** 2016-01

**Authors:** Philippe Gautret, Frank P. Mockenhaupt, Frank von Sonnenburg, Camilla Rothe, Michael Libman, Kristina Van De Winkel, Emmanuel Bottieau, Martin P. Grobusch, Davidson H. Hamer, Douglas H. Esposito, Philippe Parola, Patricia Schlagenhauf, for the GeoSentinel Surveillance Network

**Affiliations:** Institut Hospitalo-Universitaire Méditerranée Infection, Marseille, France (P. Gautret, P. Parola);; Aix Marseille Université, Marseille (P. Gautret, P. Parola);; Charité–Universitätsmedizin Berlin, Berlin, Germany (F.P. Mockenhaupt);; University of Munich, Munich, Germany (F. von Sonnenburg);; University Clinic Hamburg–Eppendorf, Hamburg, Germany (C. Rothe);; McGill University, Montreal, Quebec, Canada (M. Libman);; University Hospital, Ghent, Belgium (K. Van De Winkel);; Institute of Tropical Medicine, Antwerp, Belgium (E. Bottieau);; University of Amsterdam, Amsterdam, the Netherlands (M.P. Grobusch);; Boston University School of Public Health, Boston, Massachusetts, USA (D.H. Hamer);; Boston Medical Center, Boston (D.H. Hamer);; Centers for Disease Control and Prevention, Atlanta, Georgia, USA (D.H. Esposito);; University of Zürich Centre for Travel Medicine, Zurich, Switzerland (P. Schlagenhauf)

**Keywords:** schistosomiasis, screening, travelers, exposure, surveillance, Schistosoma, haematobium, mansoni, parasites, trematode, snail, intermediate host, urinary schistosomiasis, Bulinus truncatus snail, One Health, freshwater rivers, SCHISTO II WB IgG test, Western blot, Cavu River, Corsica, France, Italy, Europe

**In response:** We agree with Berry et al. (*1*) that the diagnostic standard for confirmation of urinary schistosomiasis is the identification of eggs by microscopic examination of urine, especially in patients living in endemic areas with high schistosome loads. However, this approach may not apply to travelers who have low parasite loads and in whom the diagnosis relies mainly on serologic testing (*2*,*3*). Given the very poor sensitivity of egg detection in non–schistosomiasis-endemic settings, most tropical and travel medicine clinics in Europe use conventional microscopy systematically combined with 2 different (commercial or in-house) serologic tests (*2*). The sensitivity of this approach (i.e., diagnosis of infection if combined ELISA and hemagglutination inhibition assay or an indirect fluorescent antibody test are positive) is >78% for chronic urinary schistosomiasis; specificity is 75%–98% when using various in-house and commercial kits (*3*). Future availability of promising ultra-sensitive tests (e.g., PCR and antigenic tests) may overcome the limitations associated with conventional microscopy and serologic testing for low-parasite load schistosomiasis.

As stated in our manuscript, we cannot exclude the possibility that our case definition generated false-positives; the potential limitations of our findings have already been discussed (*4*). Furthermore, we were cautious with our interpretation of the serologic test results and, therefore, claimed only 2 confirmed cases (*4*), 1 on the basis of egg detection and the other on positive serologic test results by using 2 different methods. We believe, on the basis of our findings (*4*) and in accordance with the European Centre for Disease Control experts (*5*), that the possibility of transmission in the Cavu River during the summer of 2014 cannot be excluded. We also want to reiterate the possibility of transmission in other rivers in Corsica, including the Solenzara, Osu, and Tarcu rivers, where *Bulinus* snails, which can serve as intermediate hosts for *Schistosoma haematobium*, were found during a malacological survey in 2014 (*5*).
